# Childhood solid tumours in relation to infections in the community in Cumbria during pregnancy and around thetime of birth

**DOI:** 10.1038/sj.bjc.6600530

**Published:** 2002-09-23

**Authors:** H O Dickinson, T A Nyari, L Parker

**Affiliations:** North of England Children's Cancer Research Unit, Department of Child Health, University of Newcastle, Royal Victoria Infirmary, Queen Victoria Road, Newcastle upon Tyne NE1 4LP, UK

**Keywords:** children cancer, solid tumours, infections, epidemiology

## Abstract

In a retrospective cohort study of all 99 976 live births in Cumbria, 1975–1992, we investigated whether higher levels of community infections during the mother's pregnancy and in early life were risk factors for solid tumours (brain/spinal and other tumours), diagnosed 1975–1993 under age 15 years. Logistic regression was used to relate risk to incidence of community infections in three prenatal and two postnatal quarters. There was an increased risk of brain/spinal tumours among children exposed around or soon after birth to higher levels of community infections, in particular measles (OR for trend=2.1, 95%CI : 1.3–3.6, *P*=0.008) and influenza (OR for exposure=3.3, 95%CI : 1.5–7.4, *P*=0.005). There was some evidence of an association between exposure to infections around and soon after birth and risk of other tumours, but this may have been a chance finding. The findings are consistent with other recent epidemiological studies suggesting brain tumours may be associated with perinatal exposure to infections.

*British Journal of Cancer* (2002) **87**, 746–750. doi:10.1038/sj.bjc.6600530 www.bjcancer.com

© 2002 Cancer Research UK

## 

Early reports of a significant association between childhood cancer and maternal viral infections during pregnancy were largely determined by childhood leukaemia (Stewart *et al*, 1958; Adelstein and Donovan, 1972; Fedrick and Alberman, 1972; Bithell *et al*, 1973). However, subsequent studies did not confirm this association and interest in this area waned (Leck and Steward, 1972; Curnen *et al*, 1974; Randolph and Heath, 1974). However, recent epidemiological studies have suggested that exposure to infections before or around birth may be associated with the risk of solid tumours, in particular brain tumours, in children (Linet *et al*, 1996; Linos *et al*, 1998; Mckinney *et al*, 1999; Fear *et al*, 2001; Mcnally *et al*, 2002). In addition, it has been suggested that JC virus has a role in the aetiology of brain tumours (Khalili, 2001).

The aim of the current study was to investigate whether, among children born in Cumbria, north-west England during 1975–92, higher levels of infection in the community around the time of birth were a risk factor for brain tumours and other solid tumours in children aged 0–14 years.

## METHODS

### Study period

A computerised database of registrations of births in the county of Cumbria from 1950–1993 (the Cumbrian Births Database) was available (Parker *et al*, 1997). However, as data on communicable diseases were available weekly for the six county districts within Cumbria only from 1975 onwards, and the World Health Organisation rule for coding the underlying cause of death changed in 1993 (Rooney and Devis, 1996), analysis was restricted to children born during 1975–92.

### The Cumbrian births database

This contains birth registration details of all births to mothers living in Cumbria, 1950–1993 (Parker *et al*, 1997). The mother's address at the time of the birth was postcoded. Date of birth and postcode were extracted for all 99 976 live births between 1975 and 1992. Births were assigned to the six county districts (average population in 1981, 79 450 (Opcs, 1983)) using digitised boundaries and the grid-reference of the postcode centroid (Local Government Act, 1972; Gregory and Southall, 1998), excluding 652 (0.7%) births for which the mother's address was missing.

### Follow-up

The children were followed up until the earliest of: end of 1993, death, emigration from the United Kingdom, diagnosis of cancer or age 15 years.

### Ascertainment of cases

Cancer registrations for the cohort, recorded throughout the UK, were obtained from the Office for National Statistics, from six regional and national cancer registries and from scrutiny of death registrations, also obtained from the Office for National Statistics (Dickinson and Parker, 1999; Dickinson *et al*, 2001). The diagnosis of all cases was reviewed centrally, from biological specimens (33%), pathology or post-mortem reports (18%), clinical records (24%) or case registration information (25%). We excluded: leukaemia and non-Hodgkin's lymphoma; retinoblastoma, as almost half the cases are hereditary (Narod *et al*, 1991); gender-specific tumours, as the population at risk differs; registrations with an ICD-O morphology code of 1 or 2 indicating non-malignancy. First malignancies were extracted. Analysis was carried out for: (i) brain and spinal tumours, (ii) other solid tumours.

### Data on infections

The numbers of cases of infections (measles, whooping cough, scarlet fever, infective jaundice and acute meningitis) were obtained from statistics on communicable diseases published for Cumbria weekly from 1975 onwards at county district level (OPCS, 1975–92) and totals were calculated for calendar months.

The numbers of deaths, at all ages, from respiratory infections (ICD 8: 460–466, 470–474, 480–486; ICD 9: 460–466, 480–487.8) in each county district were obtained from the Regional Office of the Department of Health. From these, deaths from influenza (ICD 8: 470–474, ICD 9: 487–487.8) were extracted and totals for each calendar month were calculated. Both deaths from influenza (*n*=193) and deaths from all respiratory infections (*n*=6363) were considered as exposures, since the former were rare. Since there was a change in the World Health Organisation rule for coding underlying cause of death in 1984, which resulted in a discontinuity in reported rates (Rooney and Devis, 1996), all analyses involving deaths from respiratory infections and influenza were stratified by time period: 1975–83 and 1984–92.

### Pre- and post-natal exposure periods considered

**Figure 1:**
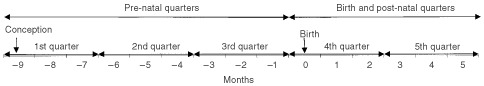
Pre- and post-natal exposure periods considered

For each birth, five periods of 3 months (quarters) were considered (see Figure 1

). Firstly, a quarter around and after birth was defined: the month of the birth and the following 2 months. The subsequent quarter and the three preceding quarters were also considered. To obtain a measure of the burden of infections in a county district in each quarter, the numbers of cases of each communicable disease and the numbers of deaths from respiratory infections in the relevant quarter were divided by the relevant inter-censal estimates of population (OPCS, 1977–94). Since deaths from influenza were rare, each birth in each quarter was classified as unexposed (no deaths from influenza in that quarter in that county district) or exposed.

### Statistical methods

As measles comprised 63% of the cases of communicable diseases, infections were aggregated into two groups: measles and other infections (whooping cough, scarlet fever, infective jaundice and acute meningitis). For measles, other infections and deaths from respiratory infections, the measure of exposure of each birth to each type of infection in each quarter was categorised into three groups (low, medium and high within each county district) using exponential grouping, which placed 64% of the births in the low group, 29% in the medium group and 7% in the high group, to achieve optimal statistical power (Connor, 1972).

As the cumulative incidence of all solid tumours increased steadily up to age 7 years and then levelled off, the age group 0–6 years was considered separately. Logistic regression was used to investigate the relationship between risk of cancer within each diagnostic group and each measure of community infections in each quarter (Hosmer and Lemeshow, 1989). The odds ratios (OR) for trend of risk across categories (low, medium and high) of community infections are reported, except for influenza where the ORs compare exposed and unexposed births. Significance was assessed by the likelihood ratio test statistic, a value of *P*<0.05 being regarded as significant. Confidence intervals were based on a quadratic approximation to the log likelihood. As other infections comprised a heterogeneous group, any significant associations between risk and exposure to these infections were further investigated by the main types of infection: whooping cough, scarlet fever, infective jaundice and acute meningitis, which comprised 58, 21, 16 and 5% respectively of this group. The goodness of fit of significant logistic models was checked using a chi-squared test. Sensitivity analysis was carried out, (a) grouping the births into three equal exposure categories within county districts and (b) grouping the births into exponential categories within all of Cumbria.

## RESULTS

The numbers of cases and incidence of solid tumours among children born in Cumbria during 1975–92 and diagnosed before the end of 1993 are shown in Table 1

**Table 1:**
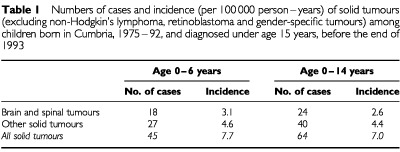
Numbers of cases and incidence (per 100 000 person–years) of solid tumours (excluding non-Hodgkin's lymphoma, retinoblastoma and gender-specific tumours) among children born in Cumbria, 1975–92, and diagnosed under age 15 years, before the end of 1993

.

### Community infections

Figure 2

**Figure 2:**
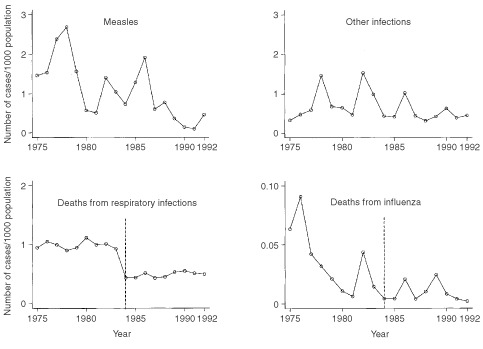
Annual incidence of community infections (number of cases/inter-censal estimate of population), for all of Cumbria, during 1975–92. (The World Health Organisation rule for coding underlying cause of death changed in 1984 (- - - - - -) (Rooney and Devis, 1996); therefore analyses involving deaths from respiratory infections and influenza were stratified by time period: 1975–83 and 1984–92.)

shows the annual incidence of the community infections, for all of Cumbria, over the time period considered. The median numbers of cases of measles and other infections per quarter in a county district were 6 (range: 0–315) and 8 (range: 0–94) respectively. The median numbers of deaths per quarter in a county district from respiratory infections and influenza were 13 (range: 0–51) and 0 (range: 0–12) respectively.

### Risk of solid tumours in relation to community infections

Table 2

**Table 2:**
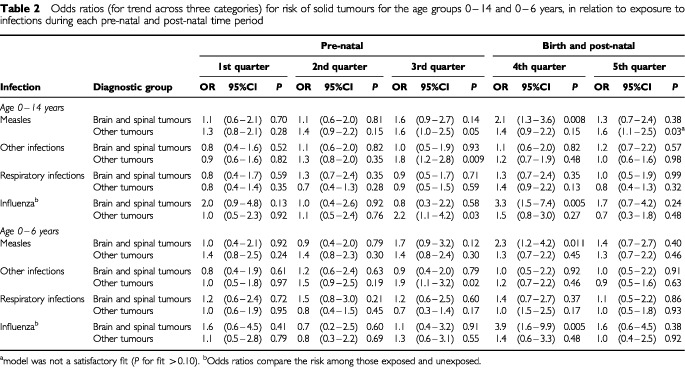
Odds ratios (for trend across three categories) for risk of solid tumours for the age groups 0–14 and 0–6 years, in relation to exposure to infections during each pre-natal and post-natal time period

shows the odds ratios for trend across three categories of risk of solid tumours in relation to the measures of exposure to measles, other infections and deaths from respiratory infections during each exposure quarter; odds ratios in relation to exposure to deaths from influenza compare two categories.

### Brain and spinal tumours

There was a significantly elevated risk of brain and spinal tumours with higher level of both measles and influenza in the fourth quarter (OR for trend=2.1, 95%CI: 1.3–3.6, *P*=0.008 and OR for exposure=3.3, 95%CI: 1.5–7.4, *P*=0.005 respectively); these effects were slightly higher in younger children. The three cases which had high exposure to measles in this quarter were all diagnosed under age 3 years, two with ependymoma and one with an unspecified brain tumour whereas, overall, 13 out of the 24 cases were astrocytomas or ependymomas. Ten of the 11 cases exposed to influenza in this quarter were all diagnosed under age 7 years, eight with astrocytoma or ependymoma and one with an unspecified brain tumour.

There was no significant association between risk and infection in any other pre- or post-natal quarter considered.

### Other solid tumours

The risk increased significantly with higher levels of other infections in the community in the third quarter (OR for trend=1.8, 95%CI: 1.2–2.8, *P*=0.009), the effect again being slightly more marked in the younger age group, where it was determined largely by exposure to whooping cough.

The risk also increased significantly with higher levels of exposure to influenza in the third quarter and to measles in the fifth quarter (although the latter model was not a good fit), but with no corresponding significant effect in the younger age group. All other significant models were an acceptable fit (*P*>0.10).

## DISCUSSION

### Strengths and weaknesses

Our study considered a longer and more recent time period than other cohort studies which have investigated the risk of solid tumours in relation to infections before or soon after birth (Fedrick and Alberman, 1972; Leck and Steward, 1972). Our ascertainment of cases was comprehensive and likely to be more complete than that of studies which did not have multiple source ascertainment; further, the diagnosis of all cases was reviewed centrally (Dickinson *et al*, 2001). Unlike other regional studies (Leck and Steward, 1972), we ascertained cases among children born in the area of interest but diagnosed elsewhere. As we estimated incidence of infection in the six county districts within the region, we had a more precisely geographically referenced estimate of exposure than other cohort studies, which assumed that all children born in the study region had the same exposure (as in the study of Leck and Steward, 1972, or the study by Fedrick and Alberman, 1972, of the risk of developing cancer among infants *in utero* during influenza epidemics). Nevertheless, some misclassification of exposure is inevitable, for several reasons. No account could be taken of urban and rural areas within county districts, although they probably have different patterns of infection; we assumed that during pregnancy mothers were living in the area where their children were born, which is unlikely to be true for all; deaths from respiratory infections and influenza will reflect the standard of clinical care as well as the prevalence of infection in the community. In contrast to case–control studies (Bithell *et al*, 1973; Linet *et al*, 1996; Mckinney *et al*, 1999; Fear *et al*, 2001) and some cohort studies (Adelstein and Donovan, 1972; Fedrick and Alberman, 1972), we did not have measures of individual exposure.

### Main findings

#### Brain and spinal tumours

We found an increased risk of brain and spinal tumours among children likely to have been exposed in the months around and just after birth to higher levels of measles and influenza. As many comparisons were made, this may be a consequence of multiple hypothesis testing. However, several factors support a genuine association: the effect was more marked in younger children and was consistent across several types of infection, and other studies have reported evidence of an infective aetiology of these tumours (Linet *et al*, 1996; Linos *et al*, 1998; Fear *et al*, 2001; Mcnally *et al*, 2002).

#### Other solid tumours

We found a significantly increased risk of other tumours (solid tumours excluding brain, spinal and gender-specific tumours, retinoblastoma and non-Hodgkin's lymphoma) among children, especially young children, likely to have been exposed in the months just before birth to higher levels of community infections other than measles; we also found a significantly increased risk among children exposed to measles 3–5 months after birth, but this effect was not significant in the younger age group. However, this heterogeneous group of tumours included many different types, which are less likely to be causally associated with the same exposure. Further, any increased risk of malignancy due to exposures around birth might be expected to be more marked in younger children, which was not always the case. Hence, since many comparisons were made, it is possible that the apparently significant findings are a chance finding due to multiple hypothesis testing.

### Comparison with other studies

#### Brain and spinal tumours

Our findings are consistent with other studies which have suggested an increased risk of brain and spinal tumours following neonatal exposure to infections. A cohort study of children born during a period of over 50 years, found space–time clustering of pilocytic astrocytoma and ependymoma in children aged 0–4 years, suggesting that pre-natal or peri-natal exposure to infection may be a risk factor for these tumours (Mcnally *et al*, 2002). Although our study did not have enough cases to analyse by specific type, the young age distribution and preponderance of astrocytomas and ependymomas among children highly exposed to peri-natal infections were consistent with the findings of McNally *et al* (2002). Some epidemiological studies have provided tentative direct evidence of the involvement of maternal viral infections in childhood brain tumours (Linos *et al*, 1998; Fear *et al*, 2001), while others have not found such an association (Bunin *et al*, 1994; Mckinney *et al*, 1999). Similarly, there is conflicting evidence about the role of neonatal infections (Linet *et al*, 1996; Mckinney *et al*, 1999). The apparently contrasting findings of these studies may be due to the differences in the infections and types of tumours studied.

Population mixing is generally regarded as precipitating a high level of infections in the community. However, using the same dataset as the present study, we found lower risks of brain and spinal tumours among children born in areas of high population mixing, which was explained by a lower risk among children of incomers (Dickinson *et al*, 2002).

#### Other solid tumours

Although a 14-fold risk of solid tumours (excluding brain and spinal tumours) has been reported among children whose mothers reported respiratory tract infections during pregnancy (Mckinney *et al*, 1999), we found no significant association between risk of these tumours and exposure to respiratory infections. This may be due to the differences between the effects of overt clinical disease and putative exposure to high levels of infections in the community.

### Conclusion

There was some evidence of an association between the risk of brain and spinal tumours and exposure to infections before and soon after birth. This supports other recent epidemiological evidence of a role of infections in the aetiology of these tumours.

## References

[bib1] AdelsteinAMDonovanJW1972 Malignant disease in children whose mothers had chickenpox, mumps, or rubella in pregnancy Br Med Jiv 629631 10.1136/bmj.4.5841.629 PMC1786989 4509453

[bib2] BithellJFDraperGJGorbachPD1973 Association between malignant disease in children and maternal virus infections Br Med Ji 706708 10.1136/bmj.1.5855.706 PMC1588846 4348514

[bib3] BuninGRCuckelyJDBoeselCPRorkeLBMeadowsAT1994 Risk factors for astrocytic glioma and primitive neuroectodermal tumor of the brain in young children: a report from the Children's Cancer Group Cancer Epidemiol Biomarkers Prev 3 197204 8019366

[bib4] ConnorRJ1972 Grouping of testing trends in categorical data J Am Stat Assoc 67 601604

[bib5] CurnenMGMVarmaAAOChristineBWTurgeonLR1974 Childhood leukaemia and maternal infectious diseases during pregnancy J Nat Cancer Inst 53 943947 4530123 10.1093/jnci/53.4.943

[bib6] DickinsonHOParkerL1999 Quantifying the effect of population mixing on childhood leukaemia risk: the Seascale cluster Br J Cancer 81 144151 10487626 10.1038/sj.bjc.6690664 PMC2374359

[bib7] DickinsonHOParkerLSalottiJBirchP2002 Paternal preconceptional irradiation, population mixing and solid tumours in the children of radiation workers Cancer Causes Control 13 183190 11936825 10.1023/a:1014384232617

[bib8] DickinsonHOSalottiJBirchPReidMMMalcolmAParkerL2001 How complete and accurate are cancer registrations notified by the National Health Service Central Register? J Epidemiol Community Health 55 414422 11351000 10.1136/jech.55.6.414 PMC1731913

[bib9] FearNTRomanEAnsellPBullD2001 Malignant neoplasms of the brain during childhood: the role of prenatal and neonatal factors (United Kingdom) Cancer Causes Control 12 443449 11545459 10.1023/a:1011201524589

[bib10] FedrickJAlbermanED1972 Reported influenza in pregnancy and subsequent cancer in the child Br Med Jii 485488 10.1136/bmj.2.5812.485 PMC1788338 4337948

[bib11] GregoryISouthallH1998 Putting the past in its place: The Great Britain historical GISInInnovations in GISCarver S (ed)London: Taylor and Francis

[bib12] HosmerDWLemeshowS1989 Applied Logistic RegressionNew York: Wiley

[bib13] KhaliliK2001 Human neurotropic JC virus and its association with brain tumours Dis markers 17 143147 11790878 10.1155/2001/423875 PMC3851256

[bib14] LeckIStewardJK1972 Incidence of neoplasms in children born after influenza epidemics Br Med Jiv 631634 10.1136/bmj.4.5841.631 PMC1786986 4674941

[bib15] LinetMSGridleyGCnattingiusSStacey NicholsonHMartinssonUGlimeliusBAdamiH-OZackM1996 Maternal and perinatal risk factors for childhood brain tumours (Sweden) Cancer Causes Controls 7 437448 10.1007/BF00052670 8813432

[bib16] LinosAKardaraMKosmidisHKatriouDHatzisCKontzoglouMKoumandakisETzartzatou-StathopoulouF1998 Reported influenza in pregnancy and childhood tumour Eur J Epidemiol 14 471475 9744679 10.1023/a:1007437200858

[bib17] Local Government Act1972Schedule 1page236London: HMSO

[bib18] McKinneyPAJuszczakEFindlayESmithKThomsonCS1999 Pre- and perinatal risk factors for childhood leukaemia and other malignancies: a Scottish case control study Br J Cancer 80 18441851 10468308 10.1038/sj.bjc.6690609 PMC2374272

[bib19] McNallyRJQCairnsDPEdenOBAlexanderFETaylorGMKelseyAMBirchJM2002 An infectious aetiology for childhood brain tumours? Evidence from space-time clustering and seasonality analyses Br J Cancer 86 10701077 11953851 10.1038/sj.bjc.6600228 PMC2364189

[bib20] NarodSAStillerCLenoirGM1991 An estimate of the heritable fraction of childhood cancer Br J Cancer 63 993999 2069856 10.1038/bjc.1991.216 PMC1972537

[bib21] OPCS1975–94 Registrar General's weekly return for England and WalesLondon: HMSO

[bib22] OPCS1977–92 Office of Population Censuses and Surveys. Vital Statistics. Series VS. Nos. 2–20London: HMSO

[bib23] OPCS1983 Office of Population Censuses and Surveys. 1981 Local authority vital statistics. Series VS. No. 8London: HMSO

[bib24] ParkerLSmithJDickinsonHBinksKScottLMcElvennyDJonesSWakefordR1997 The creation of a database of children of workers at a nuclear facility: an exercise in record linkage Appl Occup Environ Hyg 12 4045

[bib25] RandolphVLHeathCW1974 Influenza during pregnancy in relation to subsequent childhood leukaemia Am J Epidemiol 100 399409 4529579 10.1093/oxfordjournals.aje.a112051

[bib26] RooneyCDevisT1996 Mortality trends by cause of death in England and Wales 1980–94: the impact of introducing automated cause coding and related changes in 1993 Population Trends 86 2935 8987096

[bib27] StewartAWebbJHewittD1958 A survey of childhood malignancies Br Med Ji 14951508 10.1136/bmj.1.5086.1495 PMC2029590 13546604

